# Ultra-rapid and high-titer biomanufacturing of trehalose 6-phosphate by an in vitro synthetic biology platform

**DOI:** 10.1186/s40643-026-01057-w

**Published:** 2026-04-27

**Authors:** Bohua Liu, Qingqing Guo, Shuo Wang, Ting Shi, Fuping Lu, Yi-Heng P. Job Zhang

**Affiliations:** 1 College of Biotechnology, Tianjin University of Science and Technology, Tianjin, 300457 People’s Republic of China; 2 State Key Laboratory of Engineering Biology for Low-Carbon Manufacturing, Tianjin Institute of Industrial Biotechnology, Chinese Academy of Sciences, 32 West 7th Avenue, Tianjin, 300308 People’s Republic of China; 3 In Vitro Synthetic Biology Center, Tianjin Institute of Industrial Biotechnology, Chinese Academy of Sciences, 32 West 7th Avenue, Tianjin, 300308 People’s Republic of China

**Keywords:** Chemical intervention, Food security, In vitro BioTransformation, In vitro synthetic biology, Multi-enzyme molecular machine, Trehalose-6-phosphate

## Abstract

Trehalose 6-phosphate (T6P), often referred to as plant insulin, serves as a central signaling molecule that regulates carbon partitioning and sucrose flux in plants, thereby influencing key agronomic traits, such as grain yield and drought resilience. Foliar spraying of T6P has been shown to significantly enhance yield and stress tolerance of numerous grains and vegetables. To circumvent the dependency on coenzymes in natural T6P synthesis, we designed and validated an in vitro new-to-nature, coenzyme-free, minimal enzymatic pathway for the biosynthesis of T6P from maltose and polyphosphate. This three-enzyme cocktail contained maltose phosphorylase, trehalose 6-phosphate phosphorylase, and polyphosphate glucokinase, and did not involve any costly coenzymes, such as ATP or UDP. Through systematic optimization of experimental parameters (including pH, temperature, Mg²⁺, phosphate concentration, and enzyme ratios), a 93% molar yield of T6P was achieved from 10 g/L maltose. The scale-up of this in vitro bioprocess to a 100-mL bioreactor with 200 g/L maltose enabled the production of up to 541 mM T6P (i.e., 252 g/L T6P disodium salt) within two hours, corresponding to a very high volumetric productivity of 126 g/L/h. This study established a scalable and cost-competitive in vitro biomanufacturing of T6P. Chemical intervention based on timed foliar spraying of T6P to cultivated crops offers a simpler and safer agricultural practice compared to genetic modification of crops.

**Graphical abstract:**

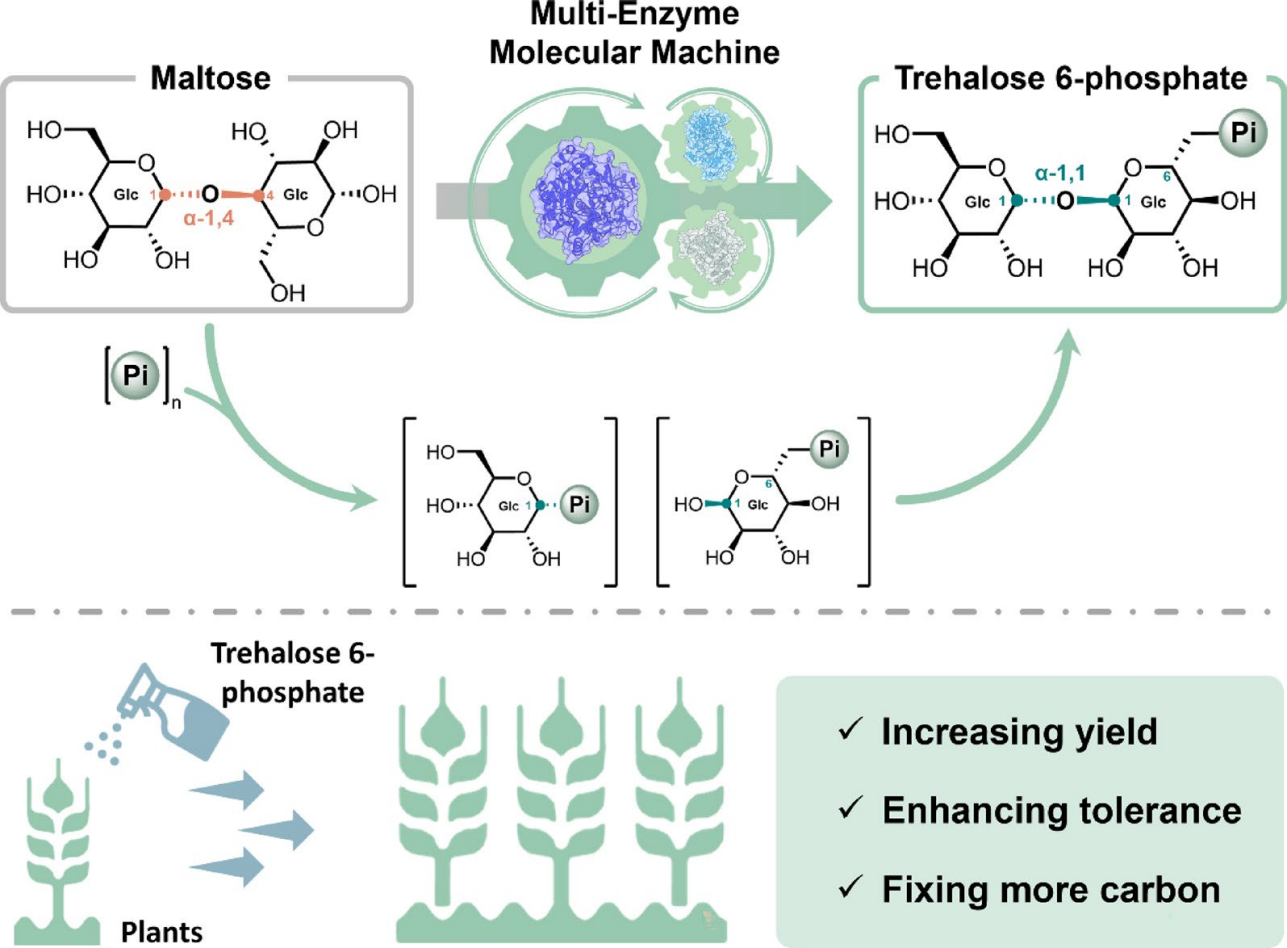

**Supplementary Information:**

The online version contains supplementary material available at 10.1186/s40643-026-01057-w.

## Introduction

**Fig. 1:**
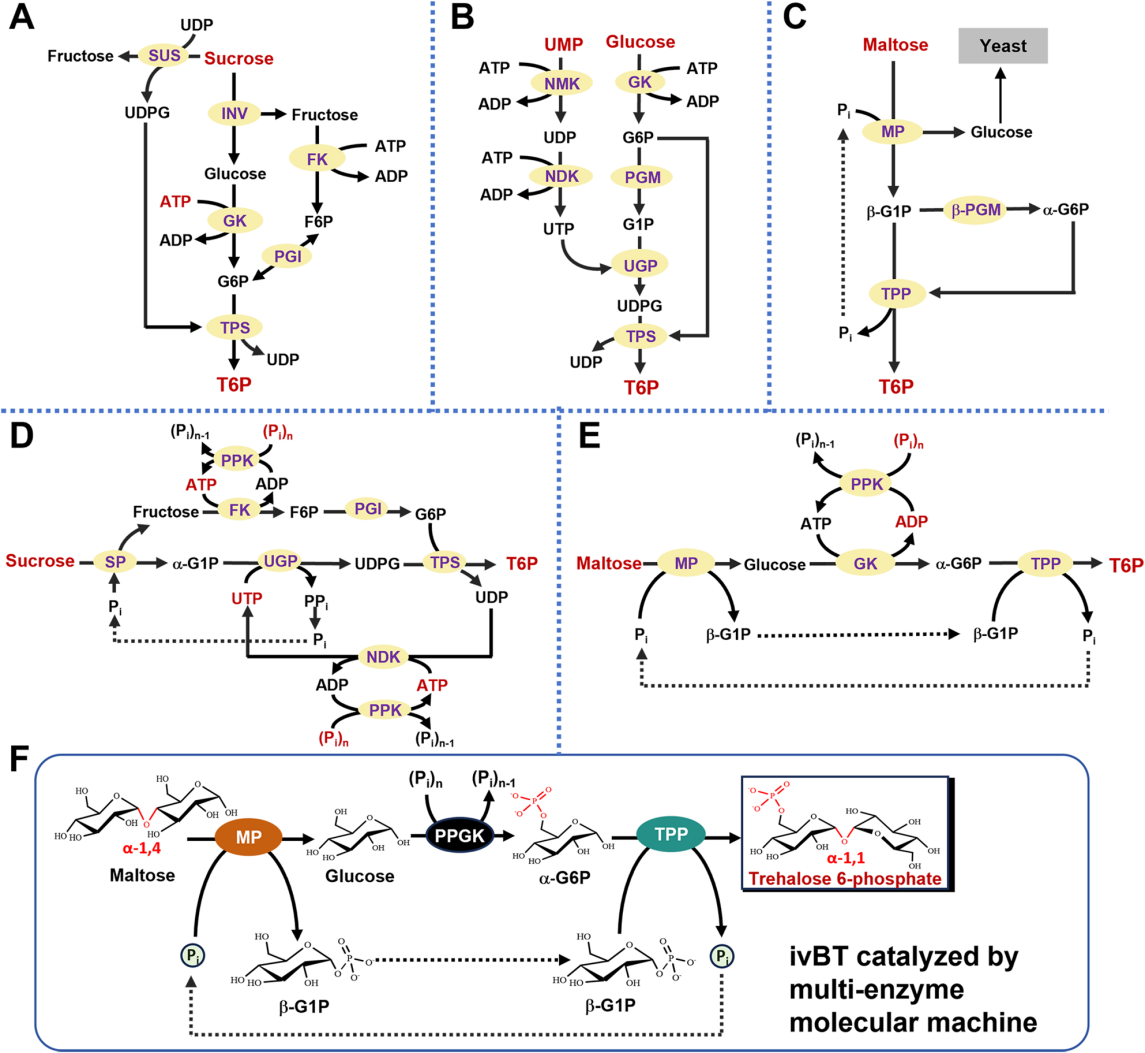
Schematic representation of the natural trehalose 6-phosphate biosynthesis pathway in plants (**A**), a specially treated living yeast system supplemented with UMP, Mg²⁺, and phosphate (**B**), a three-enzyme cocktail based on maltose in the presence of yeast to assimilate glucose (**C**), an in vitro seven-enzyme pathway based on sucrose and phosphate with the addition of UTP and ATP (**D**), an in vitro ATP-dependent four-enzyme cocktail (**E**), and an in vitro ATP-free three-enzyme cocktail (**F**) in this study. The enzymes of Pathway A are sucrose synthase (SUS), invertase (INV), glucokinase (GK), trehalose 6-phosphate synthase (TPS), fructokinase (FK), phosphoglucose isomerase (PGI). The enzymes of Pathway B are GK, phosphoglucomutase (PGM), nucleoside monophosphate kinase (NMK), nucleoside diphosphate kinase (NDK), UTP-glucose 1-phosphate uridylyltransferase (UGP), and TPS. The enzymes of Pathway C, along with yeast, are maltose phosphorylase (MP), β-PGM, and trehalose 6-phosphate phosphorylase (TPP). The enzymes of Pathway D are sucrose phosphorylase (SP), UGP, FK, PGI, NDK, polyphosphate kinase (PPK), and TPS. The enzymes of Pathway E are MP, GK, TPP, and PPK. The enzymes of Pathway F are MP, polyphosphate glucokinase (PPGK), and TPP.

Trehalose 6-phosphate (T6P) is a 6-phosphate derivative of trehalose. Trehalose is a non-reducing disaccharide in which two glucose molecules are linked together by an α,α-1,1-glycosidic bond (Fichtner and Lunn 2021; Griffiths et al. 2016; Griffiths et al. 2025). T6P is an endogenous metabolite of the trehalose biosynthesis pathway. This pathway is widely found in bacteria, fungi, plants, and invertebrates. However, it is absent in most vertebrates, particularly in mammals (Liu et al. 2025). It is typically synthesized from glucose 6-phosphate (G6P) and UDP-glucose catalyzed by trehalose 6-phosphate synthase (TPS, EC 2.4.1.15) (Fig. 1A) (Liu et al. 2025). In plants, T6P plays an important role in regulating the balanced supply and demand of sucrose from growing to sink organs (Fichtner et al. 2021). Recently, its role as a reagent of chemical intervention in agriculture was receiving wide attention because it can increase grain yield, improve crop recovery, and resurrect plants from drought (Fichtner and Lunn 2021; Griffiths et al. 2016; Griffiths et al. 2025). Therefore, it was urgently needed to synthesize a large amount of T6P with a novel cost-effective approach (Fig. 1).

Mono-saccharide phosphates can be synthesized by organic synthesis, microbial fermentation, enzymatic biocatalysis (Deng et al. 2024; Li et al. 2024a), and in vitro BioTransformation (ivBT) that can be regarded as an extension of one-pot multi-enzyme cascade reactions with advanced complexity (Chen et al. 2021; Fan et al. 2025; Li et al. 2024b; Shen et al. 2025; Zhang et al. 2023). Organic synthesis of mono-saccharide phosphates is technically mature but costly, because it requires several complicated steps of protection and deprotection of functional groups, harsh reaction conditions, low chemical selectivity, and serious pollution (Kim et al. 2016; Tasnádi et al. 2020; Wang et al. 2017; Wohlgemuth et al. 2017). Microbial fermentation of sugar phosphates was challenging because charged compounds cannot cross the cellular membrane freely. For example, fructose 1,6-bisphosphate (FBP) can be synthesized by using an organic solvent-treated living yeast, wherein organic solvents permeabilize the membrane, facilitating the FBP diffusion, but disrupt ATP synthesis ability and impair yeast viability (Wang et al. 2017). T6P has been synthesized from glucose and phosphate supplemented with UMP by using drying-treated baker’s yeast, which can continuously generate ATP by using its inherent metabolism to support this energy-intensive synthesis and allow charged compounds across the cellular membrane (Fig. 1B) (Doi et al. 1998). Therefore, enzymatic biocatalysis would be the best choice for the biosynthesis of sugar phosphates because of high selectivity and mild reaction conditions. Taguchi et al. (Taguchi et al. 2020) designed and validated an ATP-free three-enzyme cocktail that can make T6P from maltose without ATP, but half of maltose was wasted (Fig. 1C). To increase substrate utilization efficiency, sugar phosphorylation is needed, requiring ATP-dependent kinases supplemented with ATP input or costly coenzymes (Chen et al. 2021; Feng et al. 2025) (Fig. 1D and E). Recently, ivBT has been proposed to synthesize biocommodities that were large-volume but low selling-price chemicals, for example, healthy sweeteners and hydrogen, without the constraints of cell membrane or inherent metabolisms. One of the ivBT pathway design principles is based on substrate-based phosphorylation without ATP involvement (Chen et al. 2021; Fan et al. 2025; Zhang et al. 2023). However, it was very challenging to make disaccharide phosphates without ATP.

The biomanufacturing platform of in vitro synthetic biology (i.e., ivBT) or synthetic biochemistry was one of the most important biomanufacturing frontiers (Billerbeck et al. 2013; Bowie et al. 2020; Honda et al. 2016; Kwon et al. 2012; Opgenorth et al. 2017; Sperl et al. 2018; Wang et al. 2025; Zhang et al. 2023). Its core biocatalysts were comprised of in vitro synthetic enzymatic pathways for producing desired products better than living microorganisms due to numerous advantages, such as high product yield, fast reaction rate, easy process control and optimization, tolerance of toxic compounds. ivBT has been used to produce numerous monosaccharide phosphates, such as dihydroxyacetone phosphate (Billerbeck et al. 2013), FBP (Iwamoto et al. 2007; Wang et al. 2017), pentose phosphates (Wen et al. 2016), 2-deoxyribose 5-phosphate (Honda et al. 2010), xylulose 5-phosphate (Kim et al. 2016), but few for disaccharide phosphates (Feng et al. 2025; Taguchi et al. 2020; Yang et al. 2023).

Table 1 shows the experimental results of T6P biosynthesis by different methods. Figure 1A shows the natural biosynthetic pathway of T6P in plants, where the key enzyme TPS catalyzes the bioconversion of UDP-glucose and G6P to T6P and UDP (Fichtner et al. 2021). In some plant tissues, two molecules of sucrose from photosynthesis are consumed to generate one molecule each of UDP-glucose and G6P. The overall stoichiometric equation is shown in Eq. 1. It means that two molecules of sucrose along with one molecules of ATP can synthesize one molecule of T6P and two molecules of fructose with 50% carbon efficiency.

1 $${\mathrm{2}}\;{\text{sucrose + ATP }} \to {\text{ T6P + 2}}\;{\text{fructose + ADP}} $$

**Table 1:** Comparison of T6P synthesis by the enzyme cocktails

Enzymes*	Substrates^&^	Titer (g/L)	Yield (%)	Productivity (g/L/h)	References
SP, UGP, FK, PGI, NDPK, PPK, TPS	Sucrose, polyphosphate, (phosphate, ATP, UTP)	42.8	73^a^	14.3	Yang et al. (2023)
MP, TPP, β-PGM, (yeast)	Maltose, phosphate	173	38^b^	1.4	Taguchi et al. (2020)
MP, TPP, GK, PPK	Maltose, polyphosphate, (ADP)	4.2	90^b^	8.4	Feng et al. (2025)
MP, TPP, PPGK	Maltose, polyphosphate, (phosphate)	252	93^b^	126	This study

*Full names of abbreviated enzymes are given in Fig. 1

^&^Compounds in parathesis were recyclable

^a^molar yield based on sucrose; ^b^molar yield based on maltose

Special dried living yeast (i.e., air-drying followed by the desiccation under reduced pressure over phosphorus pentoxide) can be used to synthesize T6P from glucose (Doi et al. 1998) as shown in Fig. 1B. When supplemented with a large amount of UMP, Mg^2+^ and phosphate, the yeast can generate ATP from its inherent metabolism and redirect ATP toward T6P synthesis. The stoichiometric reaction (Eq. 2) suggests that two molecules of glucose along with three molecules of ATP can make one molecule of T6P with 100% carbon efficiency. Indeed, the reported molar yield was only 0.11 mol of T6P per mole of glucose (Doi et al. 1998).

2 $$ {\mathrm{2}}\;{\mathrm{Glucose}}\, + \,{\text{3 ATP }} \to {\text{ T6P}}\, + \,{\text{3 ADP}}\, + \,{\mathrm{P}}_{{\mathrm{i}}} $$

In this study, we designed and validated the new-to-nature minimized-enzyme pathway that was comprised of three enzymes [e.g., maltose phosphorylase (MP, EC 2.4.1.8), trehalose 6-phosphate phosphorylase (TPP, EC 2.4.1.216) and polyphosphate glucokinase (PPGK, EC 2.7.1.63) ] (Fig. 1F). This in vitro enzymatic pathway was independent of TPS in the natural T6P biosynthetic pathway and did not involve ATP or other costly coenzymes. This novel biomanufacturing approach would promote the wide application of chemical intervention of T6P in agriculture.

## Materials and methods

### Chemicals and strains

All chemicals used were of analytical grade or higher quality and they were purchased from Sigma-Aldrich (St. Louis, MO, USA), Aladdin (Shanghai, China), and Sinopharm (Beijing, China) unless noted. *Escherichia coli* TOP10 was used as host for DNA manipulation, and *E. coli* BL21(DE3) was used for recombinant protein expression. Lysogeny broth (LB) medium was used for *E. coli* cell growth and recombinant protein expression supplemented with 100 µg/mL ampicillin or 50 µg/mL kanamycin.

### Preparation of enzymes

The DNA sequence of MP (Uniprot ID: O06993) derived from *Bacillus subtilis* 168 was synthesized and subcloned into pET20b expression vector, yielding plasmid pET20b-BsuMP. The DNA sequence of TPP (Uniprot ID: Q9CID5) derived from *Lactococcus lactis subsp. lactis* IL1403 was synthesized and subcloned into pET28a vector, resulting in plasmid pET28a-llaTPP. Plasmid pET28a-Tfuppgk-M4-1 for the expression of an engineered polyphosphate glucokinase (PPGK) was obtained as previously described (Zhou et al. 2018). The codon-optimized DNA sequences of MP and TPP are available as On-Line Supplementary Materials.

All recombinant plasmids were transferred into *E. coli* BL21 (DE3) to express the recombinant proteins. Transformed *E. coli* cells were inoculated into LB medium containing either 50 µg/mL kanamycin or 100 µg/mL ampicillin, depending on the antibiotic resistance marker of the respective plasmid, and incubated at 220 rpm and 37 °C. When the absorbance (A600) reached at 0.8-1.0, recombinant protein expression was induced by adding isopropyl-β-D-thiogalactopyranoside (IPTG) (0.1 mM, final concentration). The cultures were incubated at 16 °C for 16 h. The cells were harvested by centrifugation at 4 °C. Then, the cells for the over-expression of MP, TPP, or PPGK were washed twice and re-suspended in 50 mM HEPES buffer (pH 7.5) containing 50 mM NaCl. The resuspended cells were lysed using high-pressure homogenization at 4 °C, and the supernatant was harvested by centrifugation at 4 °C. The target protein in the supernatant was purified by affinity adsorption on charged nickel resins. Protein concentrations were determined by the Bradford method with bovine serum albumin as a standard. The purities of recombinant proteins were checked by SDS-PAGE and analyzed by using a densitometry analysis of the Image Lab software (Bio-Rad, Hercules, CA, USA).

### Enzyme activity assays

MP activity was measured based on the generation of glucose from maltose and phosphate (Gao et al. 2019). Reaction was conducted in 50 mM sodium phosphate buffer (pH 7.5) containing 10 mM maltose and 5 mM MgCl_2_ at 37 °C for 6 min. The reaction was stopped by heating at 80 °C for 5 min. Glucose concentration in the reaction system was quantified by using a glucose oxidase-based assay kit (Applygen Technologies, Beijing, China).

TPP activity was measured in the direction of T6P synthesis, using α-glucose 6-phosphate (α-G6P) and β-glucose 1-phosphate (β-G1P) as substrates (Taguchi et al. 2020). The reaction was carried out in 50 mM HEPES buffer (pH 7.5) containing 10 mM α-G6P, 10 mM β-G1P and 5 mM MgCl_2_ at 37 °C for 3 min. The reaction was initiated by the addition of TPP and stopped by heating at 80 °C for 5 min. The released inorganic phosphate was quantified using the mild-pH phosphate assay (Saheki et al. 1985).

The activity of the *Thermobifida fusca* YX PPGK mutant was determined as described previously (Zhou et al. 2018). Its activity was measured based on the generation of G6P from 1 mM polyphosphate (i.e., 6 mM phosphate equivalents) and 5 mM glucose in a 50 mM HEPES buffer (pH 7.5) containing 4 mM MgSO_4_ at 50 °C for 15 min. The formation of G6P was then coupled to the reduction of NAD^+^ by glucose 6-phosphate dehydrogenase (0.5 U/mL) in 50 mM HEPES buffer (pH 7.5) containing 1 mM NAD⁺ at 25 °C for 10 min. The formation of NADH was monitored spectrophotometrically by measuring the increase in absorbance at 340 nm.

### Quantification of sugar phosphates and carbohydrates

T6P, G1P and G6P were quantified by using Thermo Scientific high-performance anion-exchange chromatography (HPAEC) equipped with a conductivity detector (HPAEC-CD ICS-6000, Waltham, MA, USA) and a Dionex IonPac™ AS11-HC analytical column (4 × 250 mm). The column temperature was maintained at 25 °C, the mobile phase was 10 mM KOH, and isocratic elution was performed over 30 min at a flow rate of 1.0 mL/min. The sample injection volume was 10 µL.

Glucose and maltose were quantified by HPAEC coupled with a pulsed amperometric detector (PAD) using a Dionex CarboPac™ PA20 analytical column (3 × 150 mm) at 25 °C. The flow rate was set to 0.3 mL/min and the injection volume was 25 µL. Sodium hydroxide served as the eluent, and the gradient washing was as follows: 10 mM NaOH for the first five min, a linear gradient from 10% of 100 mM NaOH to 90% of 10 mM NaOH from 5 min to 25 min, from 2% of 100 mM NaOH to 98% of 10 mM NaOH from 25 min to 26 min, followed by 100 mM NaOH from 26 min to 36 min, which was maintained until 55 min. Prior to analysis, reaction samples containing 10 g/L maltose as the substrate were diluted by 200-fold, while those containing 200 g/L maltose as the substrate were diluted by 4000-fold prior to ensure analyte concentrations fell within the linear detection range of the instrument.

### Optimization of reaction conditions

The reaction mixture containing 10 g/L maltose, 15 mM sodium hexametaphosphate (i.e., 90 mM phosphate equivalents), 20 mM sodium phosphate buffer (pH 7.5), 15 mM MgSO₄, and 0.2 g/L MP, 0.02 g/L TPP, and 0.02 g/L PPGK was incubated at 37 °C. To determine the optimal reaction parameters, reaction conditions were systematically evaluated. The optimal pH was tested from pH 6.0 to 8.0 at 37 °C for one hour. Temperature optimization was performed at pH 7.0 using temperatures of 20, 30, 37, 45, and 55 °C. The sodium phosphate buffer concentrations at pH 7.0 were tested from 10 to 100 mM at 37 °C for one hour. The concentration of sodium hexametaphosphate varied from 5 to 50 mM. The magnesium concentration was optimized from 5 to 100 mM under reaction conditions containing 20 mM sodium phosphate buffer, 15 mM sodium hexametaphosphate, pH 7.0, and 37 °C.

To determine the optimal enzyme ratio for T6P production, a ternary optimization approach was employed using the three enzymes of MP, TPP, and PPGK. A total of nine enzyme mixtures were prepared, with the total protein concentration fixed at 0.3 g/L in all cases. Each corner of the triangle represented one of the three enzymes at 100% without the other two components. Six initial combinations were selected at the intersection points of parallel lines spaced at 20% intervals along each side of the triangle, corresponding to defined proportions of the three enzymes based on barycentric coordinates. Then, three extreme combinations were added to explore boundary conditions, in which one enzyme constituted 80% of the total protein (0.24 g/L), while the other two each contributed 10% (0.03 g/L), yielding a weight ratio of 8:1:1 (MP: TPP: PPGK). For each of the nine formulations, the corresponding enzyme mixture was added to the standard reaction system, and T6P titers were quantified by HPAEC.

### Biosynthesis of high-titer T6P in 100-mL bioreactor

An enzyme cocktail of 4.8 g/L MP, 0.6 g/L TPP, and 0.6 g/L PPGK (weight ratio MP: TPP: PPGK = 8:1:1) was employed to convert 200 g/L maltose into T6P. The reaction was carried out at 37 °C in 100 mM sodium phosphate buffer (pH 7.0) containing 200 mM sodium hexametaphosphate (1,200 mM phosphate equivalents) and 75 mM MgSO_4_ on a 100-mL bioreactor. The concentration of T6P was determined as described above. To evaluate the impact of enzyme loading on T6P production, the total protein concentration of the enzyme cocktail at the MP: TPP: PPGK mass ratio of 8:1:1 was systematically decreased from 6.0 to 0.44 g/L under the same reaction conditions.

## Results

### Design of an ATP-free enzymatic pathway for T6P biosynthesis

Different from natural TPS-dependent pathways (Fig. 1A, B&D), ATP-free TPP-based enzymatic pathway (Fig. 1C), and ATP-dependent TPP-based enzymatic pathway (Fig. 1E), we designed a novel ATP-free TPP-based enzymatic pathway without carbon loss (Fig. 1F). This reaction (Eq. 3) has a Gibbs free energy of -16.4 ± 4.5 kJ/mol (http://equilibrator.weizmann.ac.il/) and a high equilibrium constant of 760, implying very high yields of T6P. To enhance the carbon efficiency, maltose was chosen as the starting substrate. MP can catalyze maltose and phosphate to generate β-G1P and glucose. Then we chose PPGK to catalyze glucose and polyphosphate to α-G6P. It was noted that PPGK can generate α-G6P from glucose and polyphosphate without requiring ATP or an ATP regeneration system (Chen et al. 2021; Zhou et al. 2018). The final step catalyzed by TPP was nearly irreversible under the standard reaction conditions, driving the overall enzymatic cascade reactions toward high titers of T6P. It was estimated that this ATP-free enzymatic pathway had a very high carbon efficiency. The combination of these three enzymatic reaction results in the stoichiometric reaction shown in Eq. 3.

3 $$ {\text{Maltose }} + {\text{ }}({\mathrm{P}}_{{\mathrm{i}}} )_{{\mathrm{n}}} \to {\text{ T6P }} + {\text{ }}({\mathrm{P}}_{{\mathrm{i}}} )_{{n - {\mathrm{1}}}} $$

### Enzyme expression and characterization

**Fig. 2:**
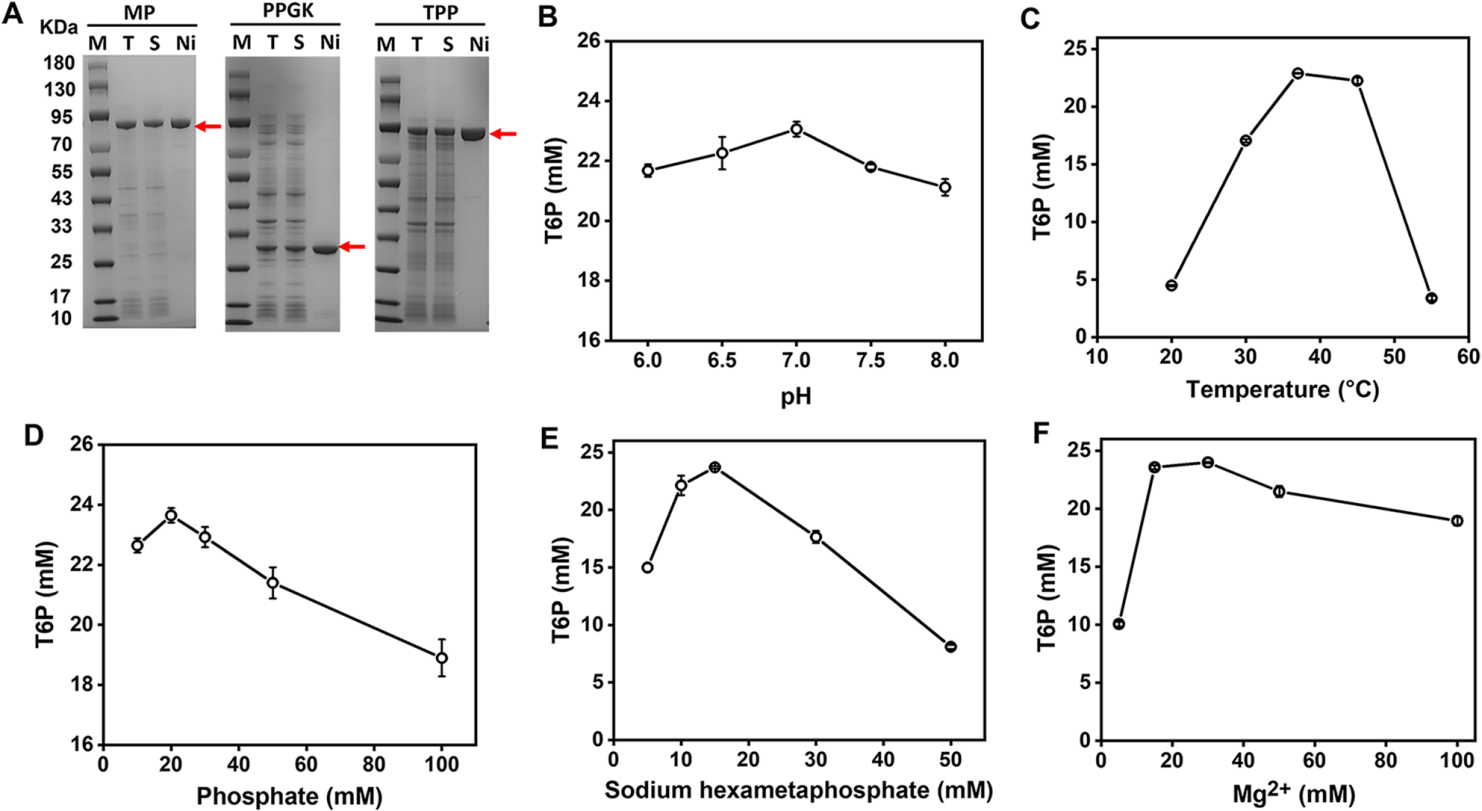
SDS-PAGE analysis of recombinant enzyme expression in *E. coli* and purification of MP, PPGK and TPP (**A**) as well as effects of pH (**B**), temperature (**C**), phosphate concentration (**D**), sodium hexametaphosphate concentration (**E**), and Mg^2+^ concentration (**F**) on T6P synthesis catalyzed by the three-enzyme cocktail (i.e., 0.2 g/L MP, 0.02 g/L TPP, and 0.02 g/L PPGK) for the one-hour reaction. M, marker; T, total cell lysate; S, supernatant; Ni, purified protein by affinity adsorption of nickel-charged resins.

According to literature reported enzymes’ data in Brenda (https://www.brenda-enzymes.org/), we carefully chose MP from *B. subtilis* and TPP from *Lactococcus lactis* due to their high specific activities. The thermostability and catalytic activity of PPGK mutants were significantly enhanced by directed evolution as described previously (Zhou et al. 2018). The three recombinant enzymes were overexpressed in *E. coli* BL21(DE3) and analyzed by SDS-PAGE (Fig. 2A). They were subsequently purified using affinity adsorption on nickel-charged resins. The specific activities of MP, TPP and PPGK were 7.5 U/mg, 109 U/mg, and 104 U/mg, respectively, at 37 °C.

### Proof-of-concept experiments and optimization

**Fig. 3:**
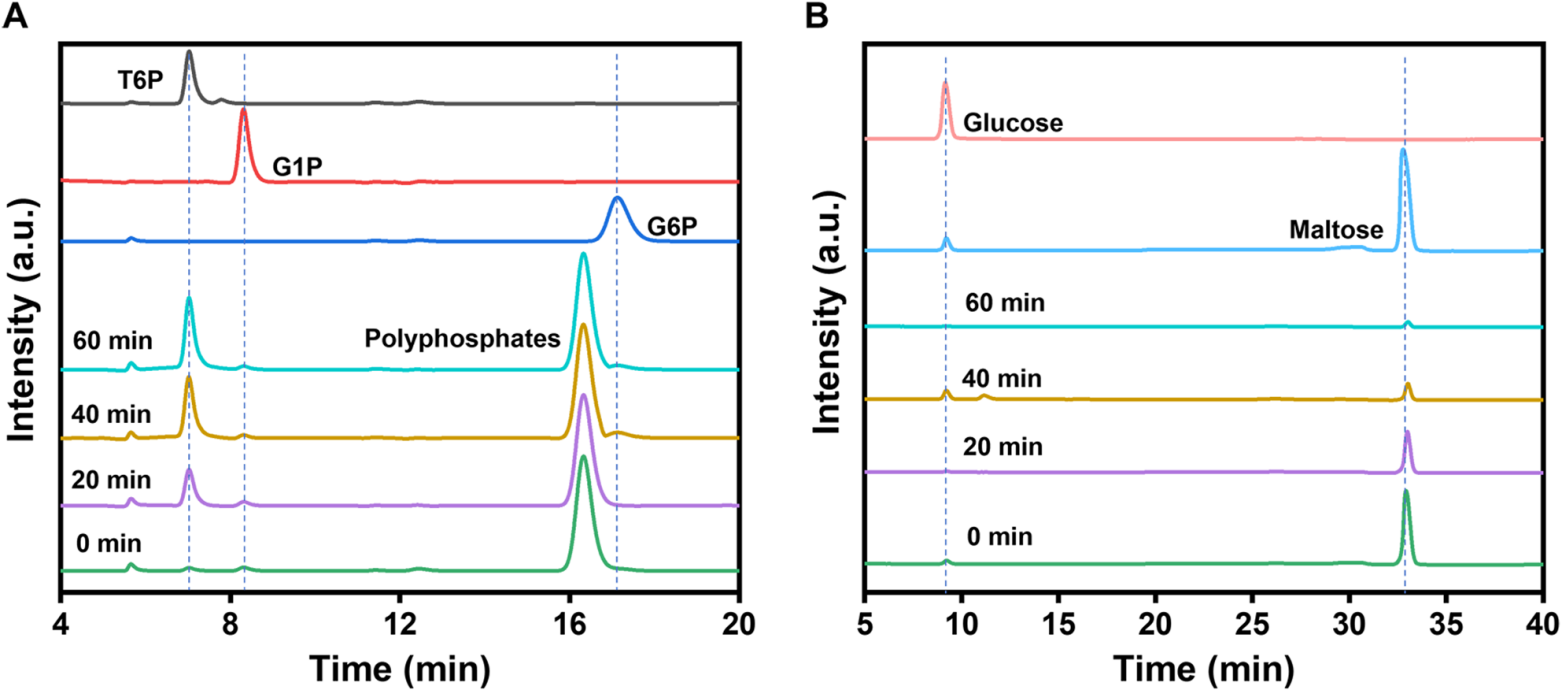
HPAEC chromatograms equipped with Dionex IonPac AS11-HC column and a conductivity detector for three standards (i.e., 0.05 g/L T6P, 0.05 g/L G1P, and 0.05 g/L G6P), and the samples after 0, 20, 40 and 60 min reaction (**A**) as well as HPAEC chromatograms equipped with the Dionex CarboPac PA20 column and a pulsed amperometric detector for 0.1 g/L glucose and 0.1 g/L maltose standards, as well as the samples after 0, 20, 40 and 60 min reaction (**B**).

HPAEC equipped with the Dionex IonPac AS11-HC column was used to measure the concentrations of sugar phosphates (i.e., T6P, G1P and G6P) with their respective retention times of 7.02, 8.32, and 17.23 min, respectively (Fig. 3A). HPAEC with the Dionex CarboPac PA20 column was used to determine the concentrations of glucose and maltose with their respective retention times of 9.09 and 33.24 min, respectively (Fig. 3B).

The proof-of-concept experiment was conducted at pH 7.5 and 37 °C using approximately equal enzyme loadings of MP, TPP, PPGK in terms of units per liter. After 60 min of the reaction, the T6P concentration reached 21.8 mM (10.2 g/L), corresponding to a molar yield of 74.6% based on the initial maltose concentration of 29.2 mM (10 g/L).

The reaction conditions for the three-enzyme cocktail were systematically optimized (Fig. 2B–F). First, the optimal pH was determined to be 7.0 (Fig. 2B), and the optimal temperature was 37 °C (Fig. 2C). Second, a phosphate concentration of 20 mM was selected as optimal due to its dual role: serving as a co-substrate for MP and as a product of the TPP-catalyzed reaction (Fig. 2D). Furthermore, the optimal substrate sodium hexametaphosphate concentration was identified as 15 mM (Fig. 2E). Lastly, magnesium ions, essential cofactors for all three enzymes and known to form complexes with phosphate that may reduce free Mg^2+^ availability, were optimized to be 30 mM (Fig. 2F).

Under the optimal conditions (i.e., 37 °C, pH 7.0, 20 mM sodium phosphate buffer, 15 mM hexametaphosphate, 30 mM Mg^2+^, and 10 g/L maltose (29.2 mM), this enzyme cocktail produced a T6P tite*r* of 24.0 mM (11.2 g/L), corresponding to a molar yield of 82.2%.

**Fig. 4:**
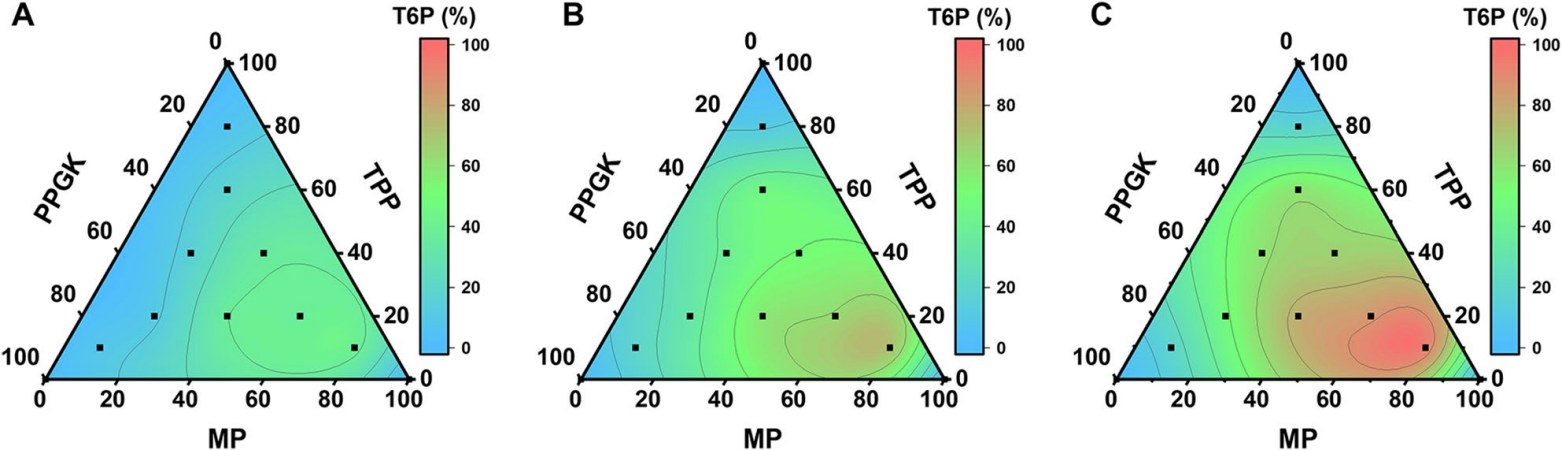
Effects of enzyme ratios on T6P synthesis. Contour plots of T6P titers at 20 min (**A**), 40 min (**B**), and 60 min (**C**) by the three-enzyme cocktail with a fixed total enzyme concentration of 0.3 g/L with different ratios of MP, TPP, and PPGK.

The enzyme ratio was further optimized by using ternary contour plot analysis on 10 g/L maltose (Fig. 4). Regardless of reaction time, the highest T6P production was obtained when the optimal composition of the enzyme cocktail was 0.24 g/L MP, 0.03 g/L TPP, and 0.03 g/L PPGK. Based on their respective molecular masses of 88.1 kD, 87.3 kD, and 43.5 kD, the molar concentrations of MP, TPP, and PPGK were 2.72 µmol/L, 0.34 µmol/L, and 0.69 µmol/L, respectively.

**Fig. 5:**
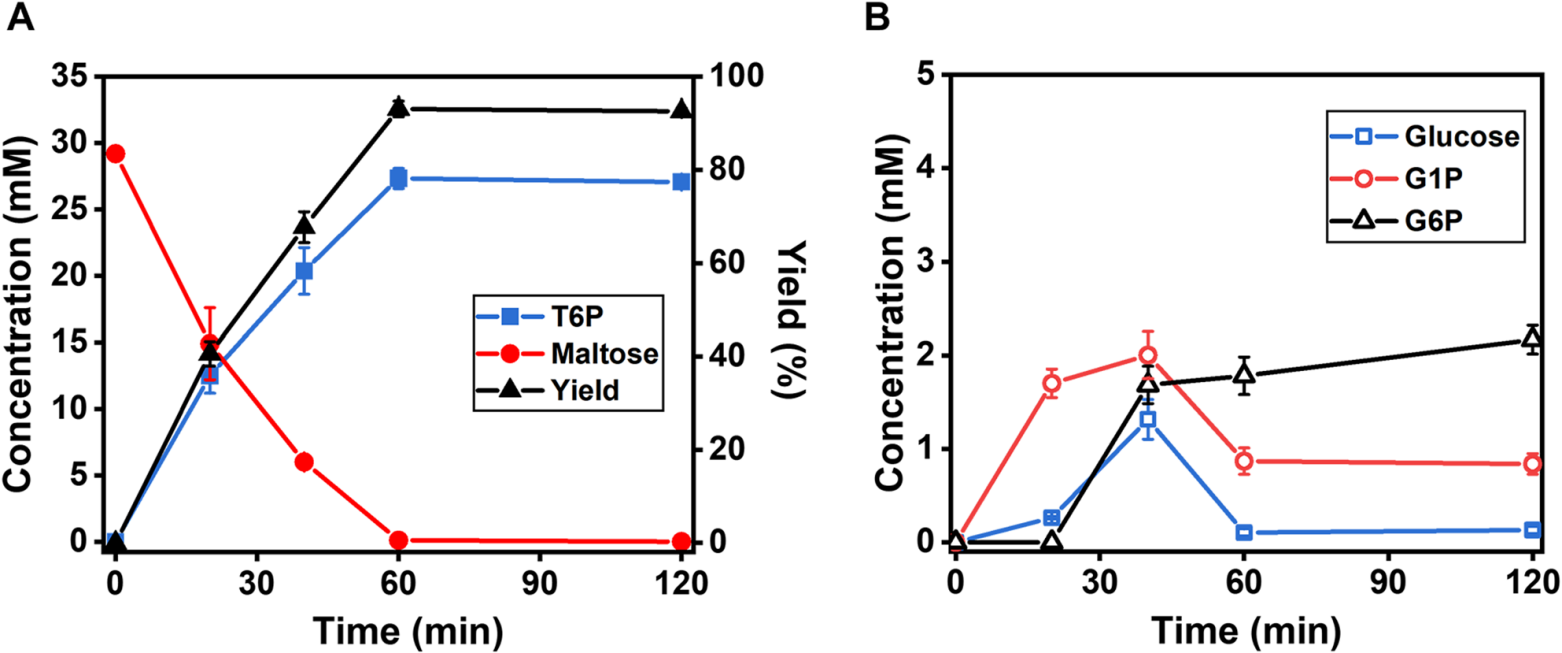
The profiles of T6P synthesis from 10 g/L maltose (**A**) as well as concentrations of intermediate metabolites G1P, G6P, and glucose (**B**).

The enzyme cocktail with the MP: TTP: PPGK mass ratio of 8:1:1 produced up to 27.2 mM T6P (Fig. 5A). Figure 5A shows that the T6P titer increased steadily over 60 min. Concurrently, maltose concentration gradually decreased (Fig. 5A). Under the optimal conditions, the molar yield of T6P reached 93.1%, representing a significant improvement over the initial proof-of-concept yield of 74.6%. Minor glucose peaks were detected, indicating low levels of glucose, likely due to trace hydrolysis of maltose (Fig. 5B).

### High-titer T6P synthesis

To demonstrate the simple and predictable scalability of the ivBT platform for the biomanufacturing of T6P, we scaled up the reaction volume from 1-mL test tube to 100-mL bioreactor where maltose concentration was increased from 10 g/L to 200 g/L (584 mM) by a factor of 20. According to the ivBT scale-up rules – using the same substrate to enzyme weight ratio, the total enzyme mass concentration was increased to 6.0 g/L by 20-fold. In principle, another substrate polyphosphate could be increased to 300 mM by 20-fold. However, we decreased its initial concentration to 200 mM because it was enough to match 584 mM maltose. To ensure a rapid utilization of 200 mM maltose, we increased phosphate to 100 mM by five-fold. Because polyphosphate and phosphate could chelate magnesium ions or form water-insoluble precipitates, leading to insufficient magnesium ions which were activators of some enzymes, we increased magnesium ions to 75 mM by 2.5-fold.

**Fig. 6:**
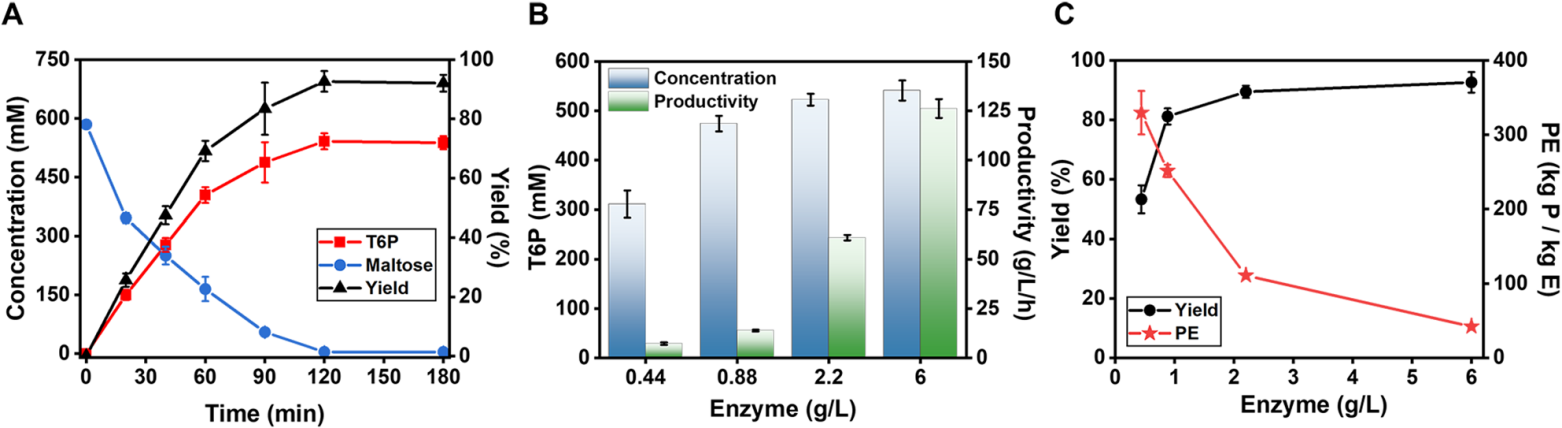
The profile of T6P synthesis from 200 g/L maltose catalyzed by a total enzyme loading of 6.0 g/L (**A**), the relationships of titer and productivity of T6P (**B**) as well as the yield and PE values (**C**) in terms of the total enzyme concentrations.

Figure 6A presents the time course of T6P synthesis on 200 mM maltose in a 100-mL bioreactor. Maltose concentration decreased over time, reaching no detectable level after 120-minute reaction. Concurrently, T6P titer increased steadily to a very high titer of 541 T6P mM (i.e., 252 g/L T6P disodium salt at pH7.5). This value corresponded to a molar conversion yield of 93%, demonstrating the high efficacy, robustness, and scalability of the ivBT system.

In addition to the TRY criteria [Titer, Rate (Productivity) and Yield] for evaluating industrial biomanufacturing, we proposed a new concept “Product-to-Enzyme Ratio” (abbreviated as PE value) that was defined as the weight ratio of the product to the weight of the lumped enzymes (Zhang et al. 2025). This PE value could serve as an industrial indicator for the economics feasibility of ivBT platform. Higher PE ratios reflected lower biocatalyst cost per unit of product.

When the total protein loading was decreased from 6.0 g/L to 0.44 g/L, the T6P titer decreased from 252 g/L to 145 g/L, while the yield declined from 93% to 53.2% and the productivity decreased from 126 g/L/h to 7.3 g/L/h (Fig. 6B and C). Although T6P titers decreased with less enzyme loading, the PE value increased from 42.1 to 330, indicating enhanced biocatalyst efficiency. These results reveal a trade-off between product titer and enzyme efficiency. At the lowest enzyme loading (0.44 g/L), the PE value reached 330, indicating that 330 g of T6P were produced per gram of the lumped enzymes. This PE value highlighted the great potential for cost-competitive biomanufacturing based on the ivBT platform (Zhang et al. 2025).

## Discussion

The in vitro enzymatic pathway developed in this study offered five distinct advantages: (i) no costly coenzymes involved (e.g., neither ATP, UMP, NAD, etc.), (ii) a minimized number of enzymes used (i.e., only three), (iii) no costly substrates, (iv) 100% carbon efficiency, and (v) high product yields due to the nearly irreversible synthesis direction. In contrast, the natural T6P biosynthetic pathway has low carbon efficiencies, e.g., 50% in plants (Eq. 1), and requires both ATP input and the other coenzymes (e.g., UDP). Recently, an in vitro four-enzyme pathway was developed to produce T6P from maltose and polyphosphate but it required exogenously-added costly ATP and yielded low T6P titers (Feng et al. 2025). This new pathway not only decreased the enzyme number from four to three, but also avoided using costly ATP without an ATP regeneration system. Furthermore, we achieved a T6P titer of up to 541 mM, 60 times of that of the four-enzyme cocktail (Feng et al. 2025). Feng’s work (Feng et al. 2025) took a wrong speculation that low T6P titers were contributed to equilibrium of cascade reversible reactions. In fact, the last reaction catalyzed by TPP (Fig. 1F) was a high value of *K*_*eq*_ (i.e., 760), which can drive the overall reactions toward the formation of T6P, leading to a very high product yield (i.e., 93.1%) in this study.

The enzyme cocktail with the best mass ratio of MP: TTP: PPGK = 8:1:1 (Fig. 4), suggesting their enzyme activity loading ratio (U/L) of approximately 3:5:5. As shown in Fig. 1F, this enzymatic pathway began with maltose, which was split to glucose and β-G1P catalyzed by MP. Then, glucose was converted α-G6P catalyzed by PPGK. Finally, the dual substrates of β-G1P and α-G6P were combined together to T6P catalyzed by TPP. According to the above mechanism, the enzyme loadings of TPP and PPGK could be equal as well as both reactions catalyzed by TPP and PPGK were not rate-limiting. The balanced supplies of β-G1P and α-G6P were in good agreement with their comparably low titers when the reactions proceeded (Fig. 5B). Then the first reaction catalyzed by MP was a rate-limiting step in this branch-merge network pathway.

These experimental results were very promising based on TRY criteria, for example, a T6P titer of 252 g/L, a molar yield of 93%, and a volumetric productivity of 126 g/L/h, as compared to previous studies (Table 1). Further improvements of this approach could be carried out in several aspects, such as (i) discovery and mining of thermostable MP and TPP, which could work together with thermophilic PPGK at elevated temperatures (Fan et al. 2025; Wu et al. 2023); (ii) enzyme engineering for enhanced thermostability and specific activity (Chen et al. 2025); and (iii) enzyme co-immobilization (Han et al. 2023a, b). Inspired by recent advances in coenzyme-free enzymatic pathways for the biosynthesis of *myo*-inositol (You et al. 2017) and D-tagatose (Fan et al. 2025), we are very optimistic of the large-scale, cost-effective biomanufacturing of T6P very soon.

In plants, T6P (plant insulin) is a signal of sucrose availability, influencing plant developmental decisions that affect the flux of sucrose to flowering, embryogenesis, and shoot branching, and links the growth of sink organs to sucrose supply (Fichtner et al. 2021). Chemical intervention in agriculture (Griffiths et al. 2016) has been suggested to be simpler and safer rather than genetic modification of crops that key genes related to the biosynthesis of T6P are overexpressed (Li et al. 2022; Nuccio et al. 2015). In this study, we demonstrated the technical feasibility of low-cost biomanufacturing of T6P from less costly substrates catalyzed by the ATP-free three-enzyme cocktail. This breakthrough in the biomanufacturing of T6P would open a door to an innovative agricultural operation -- spraying T6P or its derivatives on crops at special timings by using unmanned aerial vehicles. The use of T6P would increase crop yields and enhance their resilience to harsh conditions (Griffiths et al. 2016, 2025). As compared to genetical modification of crops with the overexpression of T6P synthesis related genes (Li et al. 2022; Nuccio et al. 2015), chemical intervention of T6P could have less debate about biosafety and be of more public acceptance.

In summary, this study demonstrated the novel biosynthesis of T6P from maltose and polyphosphate by using only three enzymes without costly coenzymes. This multi-enzyme molecular machine implemented the ATP-free glycosidic bond rearrangement from α,α-1,4-glycosidic bond of maltose to α,α-1,1-glycosidic bond of T6P. The T6P titer was up to 252 g/L of T6P disodium salt and a volumetric productivity was as high as 126 g/L/h. These results suggested that large-scale, cost-effective biomanufacturing of T6P would come true within a short time, providing a new tool to help enhance the global food security.

## Supplementary Information

Below is the link to the electronic supplementary material.

Supplementary Material 1

## Data Availability

The data supporting the findings of this study are available from the corresponding author upon reasonable request.
