# Supplementary material for: Ultra-rapid and high-titer biomanufacturing of trehalose 6-phosphate by an in vitro synthetic biology platform
Source: Bioresour Bioprocess. 2026 Apr 27;13(1):57. doi: 10.1186/s40643-026-01057-w (PMC13121652; doi:10.1186/s40643-026-01057-w)
Supplement: Supplementary file 1 — Supplementary Material 1 [file 40643_2026_1057_MOESM1_ESM.docx]

**On-Line Supplementary Materials**

**Ultra-Rapid and High-Titer Biomanufacturing of Trehalose 6-Phosphate by an in vitro Synthetic Biology Platform**

**Bohua Liu,^[a,b,c]^** **Qingqing Guo,** **^[c]^ Shuo Wang, ^[c]^ Ting Shi, ^[b,c]^ Fuping Lu,*^[a]^**

**Yi-Heng P. Job Zhang*^[a][b][c]^**

[a] College of Biotechnology, Tianjin University of Science and Technology, Tianjin 300457 (P.R. China)

[b] State Key Laboratory of Engineering Biology for Low-Carbon Manufacturing, Tianjin Institute of Industrial Biotechnology, Chinese Academy of Sciences
32 West 7th Avenue, Tianjin 300308 (P.R. China)

[c] In vitro Synthetic Biology Center, Tianjin Institute of Industrial Biotechnology, Chinese Academy of Sciences, 32 West 7th Avenue, Tianjin 300308 (P.R. China)

* Corresponding authors.

Prof. Yi-Heng P. Job Zhang, Email: [zhang_xw@tib.cas.cn](mailto:zhang_xw@tib.cas.cn), ORCID: 0000-0002-4010-2250

Prof. Fuping Lu, Email: [lfp@tust.edu.cn](mailto:lfp@tust.edu.cn)

**Sequence of MP**

ATGATAAATCAGCGGTTATTTGAGATTGATGAATGGAAAATCAAAACAAATACATTTAATAAGGAGCATACACGGCTGCTGGAAAGCCTGACGTCTCTTGCCAATGGCTATATGGGGGTCAGAGGGAATTTTGAAGAAGGCTATTCAGGCGACAGTCACCAAGGCACATATATTGCAGGCGTGTGGTTCCCCGACAAAACGCGAGTAGGCTGGTGGAAAAACGGGTATCCAGAATATTTCGGAAAAGTGATCAATGCGATGAACTTTATGGGCATAGGCCTATATGTTGACGGTGAAAAAATCGATCTCCATCAAAACCCAATCGAATTATTTGAGGTAGAACTCAATATGAAAGAGGGGATTCTGCGGCGAAGCGCTGTTGTCCGCATTCAAGATAAAACCGTCAGAATCAGGTCAGAGCGGTTTCTTAGCCTTGCTGTAAAAGAACTCTGTGCGATTCATTATGAAGCGGAGTGCTTGACGGGAGATGCTGTCATTACGCTTGTTCCTTACCTGGATGGAAATGTGGCAAATGAAGATTCTAACTACCAAGAACAGTTTTGGCAGGAGGAAGCGAAAGGTGCTGATTCTCACAGCGGCCATTTAGCGGCAAAAACGATCGAAAATCCATTTGGAACACCGCGCTTCACGGTATTAGCCGCAATGGCAAACGAGACGGAGGGTTTTGTCCATGAAAGCTTTAAAACGACTGAAATGTATGTCGAGAATCGCTACAGTTATCAAACGAAGGCATCATTGAAAAAGTTTGTGATTGTCACGACTTCCCGTGATTTTCGGGAGGAAGAGCTTTTATCGAAAGCCAAGGAGCTTTTGGCGGATGTGGTAGAGAACGGCTATGAAGATGCAAAACGGAGGCACACTGATCGATGGAAGGAAAGATGGGCAAAAGCGGACATTGAGATTAAAGGAGATGAGGAGCTTCAACAAGGAATCCGCTACAATATCTTTCAGTTATTCTCGACATATTACGGGGGCGATGCCCGTTTGAATATCGGGCCGAAAGGATTTACTGGCGAGAAATATGGAGGTGCCGCATATTGGGATACTGAGGCGTACGCCGTTCCGATGTATTTGGCGACAGCCGAGCCGGAGGTGACGAAAAACCTGCTTTTGTATCGCTATCATCAGTTGGAGGCTGCCAAACGAAACGCTGCAAAATTGGGGATGAAGGGGGCACTTTATCCGATGGTGACGTTCACAGGTGATGAATGCCACAACGAATGGGAAATCACCTTTGAAGAAATTCACCGCAATGGCGCGATCTGTTATGCGATCTACAATTATATCAATTATACAGGCGACCGTAACTATATGGAAGAATACGGGATAGACGTACTTGTGGCAGTCAGCAGATTTTGGGCCGACCGTGTTCACTTCTCGAAACGAAAAAATAAGTATATGATCCATGGCGTCACAGGGCCGAATGAATACGAAAACAACGTTAACAACAATTGGTATACGAATGTCATTGCGGCTTGGACGTTGGAGTATACTCTACAAAGTCTCGAAAGTATCTCAGCGGAGAAACGCCGCCATCTGGATGTGCAGGAAGTAGAATTGGAAGTCTGGAGAGAAATCATCCAGCACATGTACTATCCATTTAGTGAAGAACTGCAAATTTTCGTTCAGCATGACACGTTCTTGGACAAAGACCTGCAAACAGTTGACGAATTAGATCCAGCGGAACGGCCTCTTTACCAGAATTGGTCATGGGACAAGATTCTCCGCTCCAATTTTATTAAGCAGGCAGATGTTCTCCAAGGCATTTATCTTTTTAATGACCGTTTTACAATGGAAGAAAAACGGCGAAATTTTGAATTTTATGAGCCGATGACTGTTCATGAATCAAGCCTATCGCCCTCTGTCCATGCGATTCTCGCAGCCGAACTCAAGCTGGAAAAGAAAGCGCTCGAATTATATAAGCGCACAGCAAGGCTTGATCTTGATAATTACAATCATGATACGGAAGAAGGCTTGCATATTACTTCAATGACGGGTAGCTGGCTGGCAATCGTTCATGGCTTTGCAGGCATGCGCACCGCGAATGAGACGCTGTCATTTGCTCCGTTTTTGCCGAAAGAATGGGACGAATATTCATTCAACATCAATTATCGAAATCGATTAATCAATGTGACGGTTGACGAAAAGCGCGTTATTTTTGAGCTTGTAAAAGGCGAGCCGCTGCACATGAACGTTTATGAGGAACCGGTTGTCCTACAGGGACGATGTGAAAGGAGAACGCCTAATGAGctcgagcaccaccaccaccaccactga

**Sequence of TPP**

ATGACCGAAAAAGATTGGATTATTCAGTATGATAAAAAAGAAGTGGGCAAACGCAGCTATGGCCAAGAAAGCCTGATGAGCCTGGGCAACGGCTATCTGGGCCTGCGCGGCGCGCCGCTGTGGAGCACCTGCAGCGATAACCATTATCCGGGCCTGTATGTGGCGGGCGTGTTTAACCGCACGAGCACCGAAGTGGCGGGCCATGATGTGATTAACGAAGATATGGTGAACTGGCCGAACCCGCAGCTGATTAAAGTGTATATTGATGGCGAACTGGTGGATTTTGAAGCGAGCGTGGAAAAACAAGCGACCATTGATTTTAAAAACGCGCTGCAGATTGAACGCTATCAAGTGAAACTGGCGAAAGGCAACCTGACCCTGGTGACCACCAAATTTGTGGACCCGATTAACTTTCATGATTTTGGCTTTGTGGGCGAAATTATTGCGGATTTTAGCTGCAAACTGCGCATTGAAACCTTTACCGATGGCAGCGTGCTGAATCAGAACGTGGAACGCTATCGCGCGTTTGATAGCAAAGAATTTGAAGTGACCAAAATTAGCAAAGGCCTGCTGGTGGCGAAAACCCGCACGAGCGAAATTGAACTGGCGATTGCGAGCAAAAGCTTTCTGAACGGCCTGGCGTTTCCGAAAATTGATAGCGAAAACGATGAAATTCTGGCGGAAGCGATTGAAATTGATCTGCAGAAAAACCAAGAAGTGCAGTTTGATAAAACCATTGTGATTGCGAGCAGCTATGAAAGCAAAAACCCGGTGGAATTTGTGTTAACCGAGCTGAGCGCGACGAGCGTGAGCAAAATTCAAGAAAACAACACCAACTATTGGGAAAAAGTGTGGAGCGATGCGGATATTGTGATTGAAAGCGATCATGAAGATCTGCAGCGCATGGTGCGCATGAACATTTTTCATATTCGCCAAGCGGCGCAGCATGGCGCGAATCAGTTTCTGGATGCGAGCGTGGGCAGCCGCGGCCTGACCGGCGAAGGCTATCGCGGCCATATTTTTTGGGATGAAATTTTTGTGCTGCCGTATTATGCGGCGAACGAACCGGAAACCGCGCGCGATCTGTTACTGTATCGCATTAACCGCCTGACCGCGGCGCAAGAAAACGCGAAAGTGGATGGCGAAAAAGGCGCGATGTTTCCGTGGCAGAGCGGCCTGATTGGCGATGAACAGAGTCAGTTTGTGCATCTGAACACCGTGAACAACGAATGGGAACCGGATAACAGCCGCCGTCAGCGCCATGTGAGCCTGGCGATTGTGTATAACCTGTGGATTTATAGTCAGCTGACCGAAGATGAAAGCATTCTGACCGATGGCGGCCTGGATCTGATTATTGAAACCACGAAATTTTGGCTGAACAAAGCGGAACTGGGCGATGATGGCCGCTATCATATCGATGGCGTGATGGGTCCGGATGAATACCACGAAGCGTATCCGGGCCAAGAAGGCGGCATTTGCGATAACGCGTATACCAACCTGATGCTGACCTGGCAGCTGAACTGGTTAACCGAACTGAGCGAAAAGGGCTTTGAAATTCCGAAAGAACTGCTGGAAAAAGCGCAGAAAGTGCGCAAAAAACTGTATCTGGATATTGATGAAAACGGCGTGATTGCGCAGTATGCGAAATATTTTGAACTGAAAGAAGTGGATTTTGCGGCGTATGAAGCGAAATATGGCGATATTCATCGCATTGATCGCCTGATGAAAGCGGAAGGCATTAGCCCGGATGAGTATCAAGTGGCGAAACAAGCGGATACCCTGATGCTGATTTATAACCTGGGCCAAGAACATGTGACCAAACTGGTGAAACAGCTGGCGTATGAACTGCCGGAAAACTGGCTGAAAGTGAACCGCGATTATTATCTGGCGCGCACCGTGCATGGCAGCACCACGAGCCGCCCGGTGTTTGCGGGCATTGATGTGAAACTGGGCGATTTTGATGAAGCGCTGGATTTTCTGATTACCGCGATTGGCAGCGATTATTATGATATTCAAGGCGGCACCACCGCGGAAGGCGTGCATATTGGCGTTATGGGCGAAACCCTGGAAGTGATTCAGAACGAATTTGCGGGCCTGAGCCTGCGCGAAGGTCAGTTTGCGATTGCGCCGTATCTGCCGAAAAGCTGGACCAAACTGAAATTTAATCAGATTTTTCGCGGCACCAAAGTGGAAATTCTGATTGAAAACGGTCAGCTGTTACTGACCGCGAGCGCGGATCTGCTGACCAAAGTGTATGATGATGAAGTGCAGCTGAAAGCGGGCGTGCAGACCAAATTTGATCTGAAACTCGAGCACCACCACCACCACCACTGA
